# Molecular Mechanisms of Muscle Weakness Associated with E173A Mutation in Tpm3.12. Troponin Ca^2+^ Sensitivity Inhibitor W7 Can Reduce the Damaging Effect of This Mutation

**DOI:** 10.3390/ijms21124421

**Published:** 2020-06-22

**Authors:** Yurii S. Borovikov, Armen O. Simonyan, Stanislava V. Avrova, Vladimir V. Sirenko, Charles S. Redwood, Olga E. Karpicheva

**Affiliations:** 1 Institute of Cytology, Russian Academy of Sciences, 4 Tikhoretsky Av., 194064 St. Petersburg, Russia; simonyan_armen@mail.ru (A.O.S.); avrova@rambler.ru (S.V.A.); sirw@mail.ru (V.V.S.); olexiya6@ya.ru (O.E.K.); 2 Radcliffe Department of Medicine, University of Oxford, John Radcliffe Hospital, Oxford OX3 9DU, UK; credwood@well.ox.ac.uk

**Keywords:** tropomyosin, mutations in tropomyosin, muscle weakness, congenital myopathy, Ca^2+^-sensitivity of myofilament, ATPase activity of myosin, troponin inhibitor W7

## Abstract

Substitution of Ala for Glu residue in position 173 of γ-tropomyosin (Tpm3.12) is associated with muscle weakness. Here we observe that this mutation increases myofilament Ca^2+^-sensitivity and inhibits in vitro actin-activated ATPase activity of myosin subfragment-1 at high Ca^2+^. In order to determine the critical conformational changes in myosin, actin and tropomyosin caused by the mutation, we used the technique of polarized fluorimetry. It was found that this mutation changes the spatial arrangement of actin monomers and myosin heads, and the position of the mutant tropomyosin on the thin filaments in muscle fibres at various mimicked stages of the ATPase cycle. At low Ca^2+^ the E173A mutant tropomyosin shifts towards the inner domains of actin at all stages of the cycle, and this is accompanied by an increase in the number of switched-on actin monomers and myosin heads strongly bound to F-actin even at relaxation. Contrarily, at high Ca^2+^ the amount of the strongly bound myosin heads slightly decreases. These changes in the balance of the strongly bound myosin heads in the ATPase cycle may underlie the occurrence of muscle weakness. W7, an inhibitor of troponin Ca^2+^-sensitivity, restores the increase in the number of myosin heads strongly bound to F-actin at high Ca^2+^ and stops their strong binding at relaxation, suggesting the possibility of using Ca^2+^-desensitizers to reduce the damaging effect of the E173A mutation on muscle fibre contractility.

## 1. Introduction

Contraction of skeletal muscle is regulated through the thin filaments, which contain actin, tropomyosin (Tpm) and troponin (TN) [1]. When the intracellular concentration of calcium changes, tropomyosin associated with actin and troponin shifts on the surface of the actin filament, opening or closing the sites for binding of the myosin heads on actin. The electrostatic nature of the actin-tropomyosin interaction and flexibility of actin and tropomyosin [2,3] can explain the dynamic displacement of tropomyosin relative to the outer and inner domains of actin (between the blocked, closed and open positions) during contraction [3,4,5,6,7]. The change in the position of the tropomyosin strands relative to the inner domains of actin is due to the difference between tropomyosin and F-actin in their bending flexibility (therefore, variation in the persistence lengths of these proteins [4,7,8]), which presumably causes an azimuthal shift of the tropomyosin strands [3,4,5,6,7,8]. When Ca^2+^ binds to troponin-C, some actin monomers change their conformation to the switched-on state [6,8], and the persistence length of the actin filament decreases [6,7,8]. At the same time, the persistence length of the tropomyosin increases, and tropomyosin moves towards the inner domains of actin [4,6], partly exposing the myosin-binding site (“closed position”) [3,5]. At low Ca^2+^, troponin-I interacts with actin [9], switching thin filaments off [5], which leads to spatial rearrangement and an increase in the persistence length of the actin filament [6,7,8]. At the same time, the persistence length of the tropomyosin decreases [6,7,8] and restricts the tropomyosin to a position close to the outer domains of actin (the “blocked position”) [5]. In this state of the thin filament (the “off” state) [5], the strong binding of myosin with actin is inhibited [1]. When the myosin heads are strongly bound to the F-actin filament, the actin monomers are switched-on, the persistence length of the actin filament decreases, and that of tropomyosin increases [6,7,8]. In this state (the “on” state), the tropomyosin strands completely expose the binding sites of F-actin to myosin and, therefore, initiate muscle contraction [5]. Recently, amino acid residues involved in tropomyosin-actin interaction were identified [10,11]. It was also found that tropomyosin can bind to the myosin head, regulating the binding of the latter to actin [12]. Consequently, tropomyosin, which is associated with both actin and troponin, is able to bind the myosin head and is also a central link in the regulation of actin-myosin interaction.

In skeletal muscle there are three main tropomyosin isoforms—α-, β- and γ-Tpm—which are encoded by the tropomyosin 1 (TPM1), TPM2 and TPM3 genes, respectively [13]. All three isoforms exist either as homodimers or heterodimers. Mutations in the TPM genes give rise to a wide spectrum of clinically, histologically and genetically variable neuromuscular and cardiac disorders [14,15]. The numerous point mutations in TPM3 gene were found in patients with such congenital pathologies as nemaline myopathy, distal arthrogryposis, congenital muscle fibre type disproportion and cap-myopathy (for reviews, see [14,16]). The E173A mutation in position 173 of γ-tropomyosin (Tpm3.12), encoded by the TPM3 gene, was detected in a 7-year-old boy with hypotonia, feeding difficulties, motor delay and scoliosis, requiring non-invasive ventilation while ambulant. Muscle biopsies showed fibre type disproportion [17]. However, the molecular mechanisms underlying the muscle fibre dysfunction caused by this mutation are unknown.

Here, we studied the effect of the E173A mutation in recombinant Tpm3.12 on actin-myosin interaction at different simulated stages of the ATPase cycle (±Ca^2+^). Actin, myosin head (S1) and tropomyosin modified by fluorescent probes were studied in the ghost muscle fibres using polarized fluorimetry, a well-established technique for this application [18,19]. The results show that tropomyosin with E173A replacement affects the proportion of the switched-on and switched-off actin monomers, the balance of myosin subfragment-1 (S1) strongly and weakly binds with F-actin, and the position of tropomyosin during the ATPase cycle. It is assumed that the E173A mutation weakens the ability of troponin-C to switch actin monomers on and activates the strong binding of the myosin heads to F-actin at low Ca^2+^ by suppressing the troponin-I ability to switch actin monomers off, as well as induces the appearance of the strongly bound myosin heads (the rigor-like myosin heads) at relaxation, which may be one of the causes of muscle weakness. The Ca^2+^-desensitizer W7 is able to attenuate the effect of this mutation.

## 2. Results and Discussion

### 2.1. The E173A Mutation in Tpm3.12 Increases Myofilament Ca^2+^-Sensitivity and Decreases the Actin-Activated ATPase Activity of S1 at High Ca^2+^

**Figure 1:**
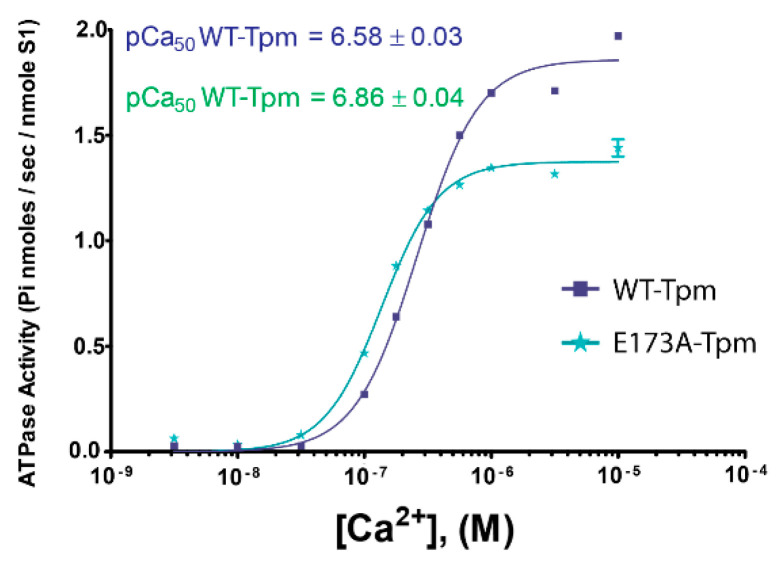
Effect of the E173A mutation in γ- tropomyosin (Tpm) on sensitivity of the thin filaments to activating Ca^2+^ concentrations. Ca^2+^-dependence was determined for fully regulated reconstituted thin filaments. The acto-S1 ATPase was measured in the presence of wild-type (WT) Tpm (squares), and E173A-Tpm (stars) at 25 °C. Error bars indicate ±SEM. pCa values were calculated from data averaged from 3 experiments. Conditions are given in Section 3.

We first evaluated the effect of E173A mutation in Tpm3.12 on Ca^2+^-sensitivity of the thin filaments reconstructed with this protein (Figure 1). The filaments were assembled with the wild-type tropomyosin (WT-Tpm) or E173A-Tpm and used in measurements of actin-activated S1 ATPase activity at increasing Ca^2+^ concentrations (see Section 3).

Measurement of actin-activated myosin S1 ATPase has revealed that the mutation increases Ca^2+^-sensitivity of the thin filaments (Figure 1). The midpoints of the curves (pCa_50_) are 6.86 ± 0.04 for filaments containing the E173A-Tpm and 6.58 ± 0.03 for those reconstituted with WT-Tpm (*p* < 0.01). In addition, the data demonstrate that ATPase rates are elevated at high pCa while reduced at low pCa and the maximum ATPase activity of S1 is lower in the presence of the mutant Tpm, than with WT-Tpm (Figure 1). An increase in myofilament Ca^2+^-sensitivity, the changes in the ATPase rates at high and low Ca^2+^ and a decrease in the actin-activated ATPase activity of S1 for the E173A-Tpm may be caused by inhibition of the ability of troponin-I to switch actin monomers off at low Ca^2+^ and by the movement of the mutant tropomyosin towards the open position at high and low Ca^2+^ (see below).

### 2.2. The Ca^2+^-Dependent Movement of Tropomyosin on the Thin Filament

**Figure 2:**
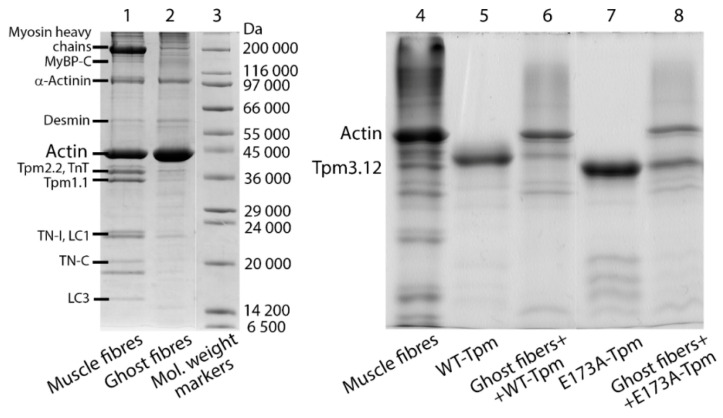
Verification of the incorporation of recombinant Tpm into the ghost muscle fibres. The SDS-PAGE shows the results of electrophoretic separation of muscle fibre isolated from *m. psoas* of rabbit (lanes 1 and 4), fibres after extraction of myosin and regulatory proteins of thin filaments (ghost fibres, lane 2), ghost fibres reconstituted with the recombinant wild-type (lane 6) and mutant (lane 8) tropomyosin incorporated in thin filaments, and the recombinant tropomyosin preparations per se (lanes 5 and 7) as well as molecular weight markers (Sigma-Aldrich, St. Louis, MO, USA, lane 3). Designations: MyBP-C—myosin-binding protein C; LC1 and LC3—myosin light chains; Tpm1.1, Tpm2.2 and Tpm3.12—tropomyosin isoforms; troponin (TN)-T, TN-I and TN-C—troponin subunits. Scanning densitometry (Image Lab 6.0) revealed that ghost fibres consist of 71% F-actin. The quantitative assessment of the Tpm binding to actin filaments in ghost fibres showed the content of the mutant Tpm bound to actin is not less than that of the WT-Tpm. Thus, the mutant Tpm can incorporate within the thin filaments in vivo. The electrophoretic mobility of the mutant Tpm differs significantly from WT-Tpm, that was already observed earlier in the case of another mutant—D175N-α-Tpm (Tpm1.1). Presumably, the less negative charge can result in different local unfolding and altered conformation. The full-length SDS-PAGE gels are given in Supplementary Materials.

Incorporation of 5-iodoacetamidofluorescein (5-IAF)-labelled recombinant wild-type γ-tropomyosin (AF-WT-Tpm) or fluorescein isothiocyanate (FITC)-phalloidin-labelled actin (FITC-actin) into the ghost muscle fibres (Figure 2) initiated polarized fluorescence. The results of fluorescence polarization measurements were fitted to the helix plus isotropic model (see Section 3).

**Figure 3:**
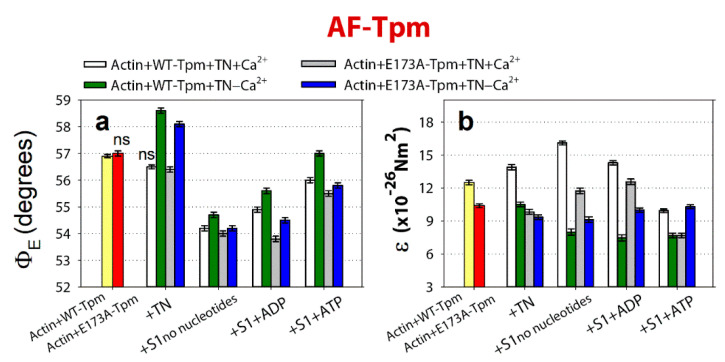
The effect of troponin and Ca^2+^ on the values of Ф_Е_ (**a**,**b**) of the polarized fluorescence for 5-iodoacetamidofluorescein (5-IAF) linked with WT-Tpm and E173A-Tpm was revealed in glycerol-skinned fibres under conditions simulating the sequential steps of the actomyosin ATPase cycle. The relative amount of the WT and mutant Tpms, as well as the amount of S1 in all experiments were monitored by measuring the fluorescence intensity of proteins in the actin-WT-Tpm-S1 and actin-E173A-Tpm-S1 complexes. When simulating rigor and in the presence of Mg-adenosine 5′-diphosphate (MgADP), the molar ratio of S1 to actin was 1:(2.4 ± 0.6) and the molar ratio of tropomyosin to actin was 1:(7.0 ± 0.5). In the presence of Mg-adenosine 5′-triphosphate (MgATP), a reduction in the molar ratio of S1 to actin was seen, which was similar for the WT and mutant Tpms (Section 3). It is supposed that the same fraction of actin monomers bound S1 in each case. The values of Ф_Е_ were corrected in order to take into account the changes in conformation of actin monomers. The first and second entries from the left in each panel present the data obtained in the absence of S1. The data represent the mean values for 5–7 fibres for each experiment (Section 3). The Ф_Е_ and ɛ values in the absence and in the presence of nucleotides are significantly altered by troponin and Ca^2+^ for both WT-Tpm and E173A-Tpm (*p* < 0.05). Error bars indicate ±SEM. The values of N were close to zero.

The values of the angle between the fibre axis and the emission dipole of the probe (Φ_E_) were 56.9° and 47.3° for AF-WT-Tpm and FITC-actin-WT-Tpm, respectively. The value of the bending stiffness (ε) was 12.5 × 10^−26^ N∙m^2^ for WT-Tpm (Figure 3b) and 5.32 × 10^−26^ N∙m^2^ for F-actin filaments containing WT-Tpm, showing that Tpm3.12 is more than two times less flexible than F-actin. Similar differences in the bending stiffness between F-actin and Tpm1.1, F-actin and Tpm2.2 or F-actin and Tpm3.12 were found earlier [7,8,20].

As shown in Figure 3, the binding of troponin to the Actin-AF-WT-Tpm complex at high Ca^2+^ results in a decrease in the values of Φ_E_ by 0.4° and an increase in ε by 1.4 × 10^−26^ N∙m^2^; whereas at low Ca^2+^ the value of Φ_E_ increases by 1.7° and the value of ε decreases by 2.0 × 10^−26^ N∙m^2^ (*p* < 0.05).

**Figure 4:**
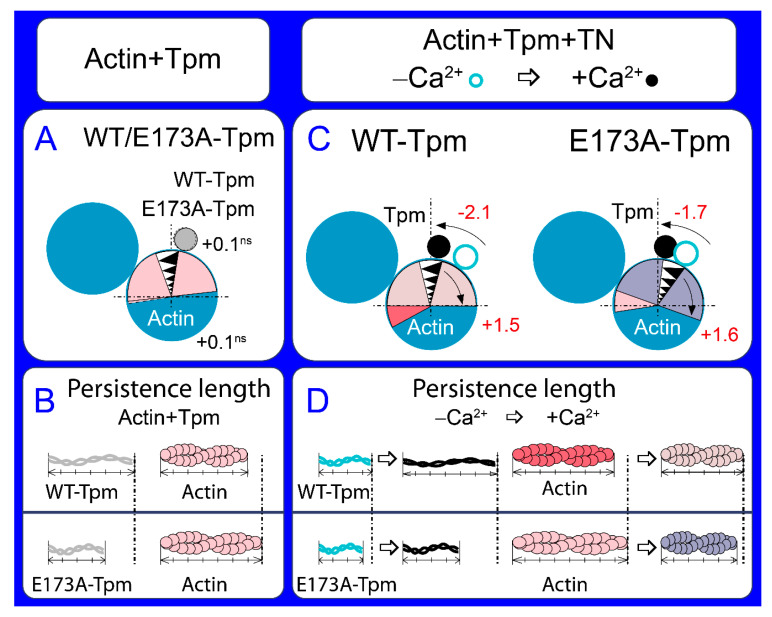
A schematic view of the changes in tropomyosin localization and spatial rearrangements of actin caused by the E173A substitution in Tpm, as follows from the data of polarized fluorimetry. The changes in the value for Φ_E_ (the angle of orientation of the emission dipoles of the probe bound to Tpm or actin monomers) are interpreted as the shift of Tpm relative to the inner and outer domains of actin and the rotation of actin monomers in actin filament. The changes in the value for bending stiffness ε are shown as the alterations in the persistence length of the Tpm strands and actin filament. Ghost fibres were composed of actin and Tpm (**A**,**B**) or of actin, Tpm and TN (**C**,**D**). (**A**) The position of the mutant Tpm and actin conformation in the region of the dye binding do not differ from that of the wild-type (WT)-Tpm in ghost fibres containing only Tpm. (**B**) However, the mutation decreases the persistence length of Tpm and increases that of actin. (**C**) The effect of the mutation emerges in the presence of troponin—at low Ca^2+^ Tpm shifts towards the inner domains of actin and actin monomers switch on. (**D**) The persistence length is lower for the mutant Tpm than for the WT-Tpm. The increase in the persistence length for WT and mutant Tpms during the rise of Ca^2+^ concentration is followed by the decrease in this parameter for actin. Designations: The changes in Φ_E_ at high and low Ca^2+^ are shown by numbers (**C**), the direction of the rearrangements is depicted by arrows. The changes in Φ_E_ (shown by numbers with symbols “ns”) are non-significant in the absence of troponin (**A**). Different conformational states of actin and localization of Tpm and respective persistence length of actin and Tpm are depicted by different colours.

The character of the changes in the Φ_E_ value for 5-IAF-labelled Tpm is correlated with the azimuthal shifting of Tpm strands observed in electron microscopy works [3,5]. An increase in the Φ_E_ value is correlated with the azimuthal shifting of Tpm strands towards the outer domains of actin subunits, while a decrease in that value is correlated with the shifting of Tpm to the inner domains [6,7,8] (Figure 4C). An increase in the flexibility of Tpm can be explained by a decrease in its persistence length [4]. Consequently, troponin at high Ca^2+^ induces a persistence lengthening of WT-Tpm strands (Figure 4D) and their shift to the inner domains of actin (Figure 4C). At low Ca^2+^ WT-Tpm was found closer to the outer domains (Figure 3a and Figure 4C).

**Figure 5:**
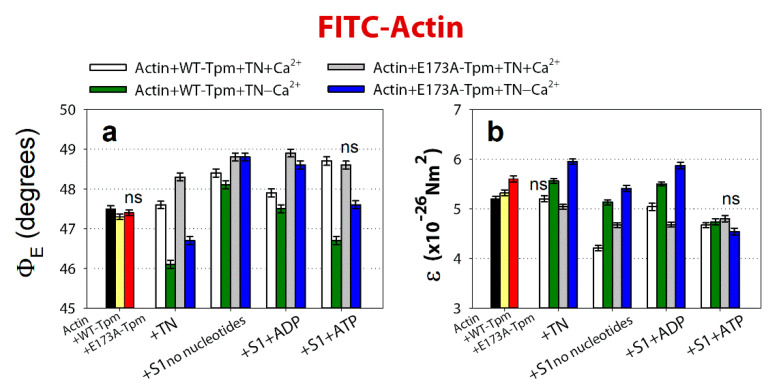
The effect of troponin and Ca^2+^ on the values of Φ_E_ (**a**) and ɛ (**b**) of the polarized fluorescence for FITC-phalloidin-actin, in the presence of WT-Tpm or E173A-Tpm under conditions simulating the sequential steps of the myosin ATPase cycle. The first and second entries from the left in each panel present the data obtained in the absence of S1. The relative amount of the WT and mutant Tpms, as well as the amount of S1 in all experiments was similar in the Actin-WT-Tpm-S1 and Actin-E173A-Tpm-S1 complexes (see Section 3), suggesting that the same fraction of actin monomers bound S1 in each case. Calculations of Φ_E_ and ɛ values, preparation of the fibres, their composition and the conditions of the experiments are described in Section 3. The data represent the mean values for 8–10 fibres for each experimental condition. Both in the absence and in the presence of nucleotides the Φ_E_ and ɛ values for WT-Tpm and E173A-Tpm are significantly altered by troponin and Ca^2+^ (*p* < 0.05). Statistically insignificant differences in the values of Φ_E_, ɛ and N between WT and mutant Tpms are indicated by the symbol “ns”. Error bars indicate ±SEM. The values of N were close to 0.1.

A similar Ca^2+^-dependent difference in azimuthal positions of WT-Tpm strands on the thin filament was observed earlier [6,7,8]. A similar correlation was observed in the presence of myosin heads (S1) at various mimicked states of the ATPase cycle (Figure 3). The movement of Tpm relative to the inner or outer actin domains correlates, respectively, with an increase and decrease in the relative number of the switched-on and switched-off actin monomers [6,7,8] in the thin filaments at the regulation of the thin filament by Ca^2+^ [2,3,4,5]. The data obtained in this work (Figure 5) correspond to these views.

### 2.3. The Ca^2+^-Dependent Switching On and Off of the Thin Filament

The binding of troponin to the FITC-Actin-WT-Tpm complex at high Ca^2+^ increases the value of Φ_E_ by 0.3° and decreases ε by 0.12 × 10^−26^ N∙m^2^ (*p* < 0.05), whereas at low Ca^2+^ the value of Φ_E_ decreases by 1.2° and the value of ε increases by 0.24 × 10^−26^ N∙m^2^ (*p* < 0.05; Figure 5). According to our earlier published works, alterations in the Φ_E_ and ε values for FITC-actin may be interpreted as a result of conformational changes (global and/or local), accompanied by switching of actin monomers on and off, respectively, which is associated with an enhancement or a reduction in the ability of F-actin [6,7,8] to activate myosin ATPase [1]. The value of ε for FITC-Actin-WT-Tpm-TN is lower at high Ca^2+^ than at low Ca^2+^ (Figure 5b). An increase and a decrease in the flexibility of the thin filaments correlate with F-actin shortening and elongation of the persistence length (Figure 4D), respectively [6,7,8]. At low Ca^2+^, troponin switches actin monomers off and induces an increase in the persistence length of F-actin (Figure 4D).

At high Ca^2+^ opposite changes occur. Similar increases and decreases in the persistence length of the thin filaments were observed by Isambert and coworkers [21] at lowering and rising Ca^2+^ concentration, respectively.

Thus, at high Ca^2+^ WT-Tpm shifts in the direction of the inner actin domains (to the closed position, [5]) and the relative number of the switched-on actin monomers increases. On the contrary, at low Ca^2+^, it shifts towards the outer domains of actin (towards the blocked position, [5]) and inhibits the switched-on actin monomers. The same Ca^2+^-dependence was observed also in the presence of myosin heads (S1) at mimicking various states of the ATPase cycle.

**Figure 6:**
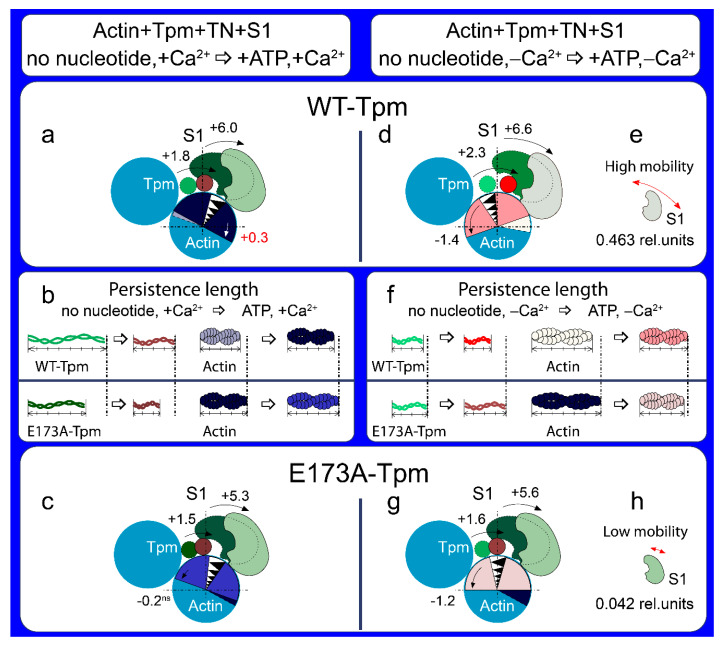
A schematic view of the changes in tropomyosin localization and spatial rearrangements of actin and S1, as follows from the data of polarized fluorimetry. The changes in the value for Φ_E_ (the angle of orientation of the emission dipoles of the probe bound to Tpm, actin monomers or the myosin heads) at simulation of different conformational states of Actin-Tpm-TN-S1 complex are interpreted as the shift of Tpm relative to the inner and outer domains of actin, the rotation of actin monomers in actin filament and the azimuthal tilt of the myosin motor domain (**a**,**c**,**d**,**g**). The left and right panels show the transition of actin-myosin from the state in the absence of nucleotide to the state in the presence of ATP at high and low Ca^2+^ concentrations, respectively. The changes in the value of bending stiffness ε are shown as the alterations in the persistence length of Tpm strands and actin filament (**b**,**f**). The effect of the E173A mutation in Tpm is discussed in the text. Briefly, the mutant Tpms shift towards the inner domains of actin, which cause the abnormal switching on of actin monomers and the appearance of the myosin heads strongly bound with actin under relaxing conditions (**a**,**c**,**d**,**g**). The persistence length of Tpm is decreased while that of actin filament is increased by the mutation at high Ca^2+^ (**b**). The mobility of the myosin heads in the presence of the mutant Tpm is decreased (**e**,**h**). Designations: The changes in Φ_E_ between the states are shown by numbers; the direction of the rearrangements is depicted by arrows. Different conformational states of actin and myosin head, changes in localization of Tpm and respective persistence lengths of actin and Tpm are depicted by different colours.

At high Ca^2+^, an increase and decrease in Φ_E_ values for FITC-actin (Figure 5) and AF-Tpm (Figure 3), respectively, were observed at the mimicked strong-binding of the myosin heads (AM and AM^∙ADP states, where A is actin, M, M^ are myosin heads in various conformational states in the absence of nucleotide or in the presence of MgADP, respectively). In the presence of MgATP (mimics AM**∙ADP∙Pi state, where A is actin, M** is myosin head in weak-binding conformational state), the values of Φ_E_ decreased for FITC-actin (Figure 5a) and increased for AF-Tpm (Figure 3a). This indicates that at high Ca^2+^ the myosin heads in the strongly-bound states cause a noticeable displacement of WT-Tpm in the direction of the inner domains of actin (a shift to the open position [5]) (Figure 6a) and significantly increase the amount of the switched-on actin monomers (Figure 5a). On the contrary, at low Ca^2+^, WT-Tpm shifts to the outer domains of actin (Figure 3a), and the amount of the switched-on actin monomers decreases (the values of Φ_E_ for FITC-actin are lower, and for AF-Tpm are higher, than at high Ca^2+^, Figure 3 and Figure 5). The E173A mutation alters this pattern (Figure 3, Figure 5 and Figure 6).

### 2.4. The E173A Mutation in the Tpm3.12. Inhibits the Ability of Troponin to Switch the Thin Filaments Off in Muscle Fibres at Low Ca^2+^

According to Figure 3, Figure 4A and Figure 5, replacing Ala for Glu residue in position 173 in tropomyosin does not have a noticeable effect on the position of AF-E173A-Tpm on the thin filament and on the amount of the switched-on actin monomers. The Ca^2+^-dependent movement of the mutant Tpm and a change in the amount of the switched-on actin monomers are observed in the presence of troponin. At high Ca^2+^, the exchange of WT-Tpm for E173A-Tpm in the Actin-Tpm-TN complex in the absence and in the presence of S1 does not change the values of Φ_E_ for AF- E173A-Tpm or decreases this value by 1.1° and by 0.5° in the presence of MgADP and MgATP, respectively, showing the shift of the E173A-Tpm towards the open position (Figure 6a,c). [5,6,7,8]. In addition, the mutation decreases the ε values at mimicked different stages of the ATPase cycle (by 4.06 × 10^−26^ N∙m^2^ in the absence of S1 and by 4.36 × 10^−26^ N∙m^2^, 1.73 × 10^−26^ N∙m^2^ and 2.25 × 10^−26^ N∙m^2^ for AM, AM^∙ADP and AM**∙ADP∙Pi states, respectively) (Figure 3a,b). A similar Tpm movement is observed at low Ca^2+^ (the values for Φ_E_ decrease at mimicking all states of the ATPase states (Figure 3a,b), but the values for ε increase. Consequently, practically at all states of the ATPase cycle the E173A substitution can change actin-myosin interaction and ATPase rates (Figure 1) in a way that is consistent with movement of the mutant Tpm strands towards the inner domains of actin both at high and low Ca^2+^, as compared with WT-Tpm (Figure 6a,e).

Such changes in the Tpm position at low Ca^2+^ can have a significant impact on the ability of troponin to regulate the actin-myosin interaction, because instead of E173A-Tpm’s shift towards the blocked position and a reduction in the amount of the switched-on actin monomers one can observe the movement of Tpm to the open position (Figure 3 and Figure 6) and an essential increase in the amount of the switched-on actin monomers at all stages of the ATPase cycle (Figure 5). Consequently, the exchange of WT-Tpm for E173A-Tpm can impair the regulatory function of thin filaments—the ability of troponin to shift tropomyosin towards the blocked position, to switch actin monomers off. Therefore, in the presence of E173A-Tpm the inhibition of the strong binding of the myosin heads to actin cannot be reached at low Ca^2+^ (see below).

### 2.5. The Ca^2+^-Dependent Formation of the Strong and Weak Binding of the Myosin Heads to F-Actin

**Figure 7:**
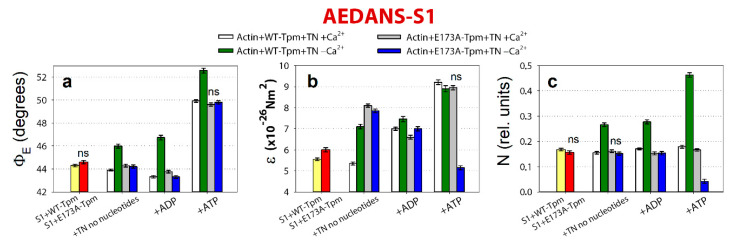
The effect of troponin and Ca^2+^ on the values of Φ_E_ (**a**), ɛ (**b**) and N (**c**) of the polarized fluorescence of N-(iodoacetaminoethyl)-1-naphthyl-amine-5-sulfonic acid (1,5-IAEDANS) bound to S1 (AEDANS-S1), in the presence of WT-Tpm or E173A-Tpm was revealed in glycerol-skinned fibres under conditions simulating the sequential steps of the actomyosin ATPase cycle. The data represent the mean values for 5–7 fibres for each experimental condition. The Φ_E_, ɛ and N values in the absence of troponin and in the presence of Ca^2+^ and nucleotides are significantly altered by the E173A mutation in Tpm (*p* < 0.05). Statistically insignificant differences in the values of Φ_E_, ɛ and N between the WT and mutant Tpms are indicated by the symbol “ns”. Error bars indicate ±SEM.

According to Figure 7, for the Actin-WT-Tpm-AEDANS-S1 complex, the values for the angle between the fibre axis and the emission dipole of the probe (Φ_E_), the value of N and the bending stiffness (ε) were found to be equal to 44.3°, 0.168 rel. units and 5.55 × 10^−26^ N∙m^2^, respectively. This indicated that the probes are highly oriented, and the myosin heads are bound strongly to F-actin [6,7,8]. Since AEDANS was rigidly bound to S1, it was assumed that the value of ε contains information about the bending stiffness of the F-actin filaments in the region of localization of the myosin heads, whereas the parameter N estimates the flexibility of attachment of the myosin heads to F-actin [22]. The bending stiffness of F-actin filaments which was determined using the polarized fluorescence of FITC-phalloidin (Figure 5b) did not differ much from that for F-actin in the areas of localization of myosin heads under all experimental conditions (Figure 7b). This observation demonstrates the possibility of a transition of the changes in actin monomer conformation along the thin filament. Transition of the signal along the thin filament was previously shown by Barua [11].

The binding of troponin to Actin-WT-Tpm-AEDANS-S1 complex induces a change in the values of Φ_E_, ε and N: at high Ca^2+^ they decrease by 0.4°, 0.2 × 10^−26^ N∙m^2^ and 0.02 rel. units (*p* < 0.05), respectively (Figure 7). Previously a correlation was found between the parameters Φ_E_ and *N* of polarized fluorescence for S1 and the affinity of the myosin head attachment to actin. It turned out that a decrease in the values of Φ_E_ and N correlates with an increase in the affinity of myosin for actin; on the contrary, an increase in these parameters is observed in parallel with a decrease in myosin’s affinity for actin [23]. Based on this correlation, we assume that a decrease in the angle Φ_E_ indicates the formation of a stronger form of actin-myosin binding, and an increase in this parameter, on the contrary, indicates the formation of a weaker form. Therefore, the changes in the values of these parameters can be interpreted as showing an increase in the number of myosin heads strongly bound to F-actin in the ghost muscle fibres [7,8,22]. On the contrary, at low Ca^2+^ these parameters essentially increase by 1.7°, 1.55 × 10^−26^ N∙m^2^ and 0.098 rel. units (*p* < 0.05), respectively (Figure 7). Thus, WT-Tpm-TN complex at high Ca^2+^ is able to facilitate, and at low Ca^2+^ to inhibit, the strong binding of the myosin heads to the thin filaments [6].

A similar pattern of changes at high Ca^2+^ is also observed at the mimicked AM^∙ADP and AM*∙ADP∙Pi states of the ATPase cycle (Figure 6a and Figure 7). In the presence of MgADP the nucleotide activates strong binding of the myosin heads to F-actin; the values of Φ_E_ are smaller and ε and N are higher than in the absence of the nucleotide. In the presence of MgATP a decrease in the number of myosin heads strongly bound to actin is observed (the values of Φ_E_, ε and N are higher than in the presence of MgADP, Figure 7). At low Ca^2+^, the number of myosin heads strongly bound to F-actin is dramatically decreased (the values of Φ_E_, ε and N increase by 3.4°, 0.46 × 10^−26^ N∙m^2^ and 0.11 rel. units, respectively, in the presence of MgADP, and the values of Φ_E_ and N increase by 2.65° and 0.284 rel. units, respectively, in the presence of MgATP, Figure 7). Consequently, upon mimicking the strong-binding states of the ATPase cycle, WT-Tpm locates close to the open position (Figure 3a and Figure 6a); the amount of the switched-on actin monomers (Figure 5) and strongly bound myosin heads (Figure 7) is higher than upon mimicking the weak-binding state. At low Ca^2+^ WT-Tpm moves towards the blocked position (Figure 3a); the amount of the switched-on actin monomers (Figure 5) and the myosin heads strongly bound to actin decreases (Figure 5 and Figure 7). The E173A mutation alters this picture (see below).

### 2.6. E173A Mutation Inhibits the Strong Binding of the Myosin Heads to F-Actin at High Ca^2+^ and Activates It at Low Ca^2+^

As opposed to the Actin-WT-Tpm-TN-AEDANS-S1 complex, the complex where WT-Tpm was replaced by the E173A-Tpm shows at low Ca^2+^ an increase in the number of the myosin heads, strongly bound to F-actin (in the absence of nucleotide the Φ_E_ and N values are lower for E173A-Tpm than for WT-Tpm by 1.8° and 0.114 rel. units, respectively (*p* < 0.05; Figure 7)).

A similar increase in the number of myosin heads strongly bound to actin at low Ca^2+^ was observed also in the presence of MgADP and MgATP (the mimicked AM^∙ADP and AM**∙ADP∙Pi states). In these cases, the parameters Φ_E_, ε and N are reduced in the presence of MgADP by 3.4°, 0.46 × 10^−26^ N∙m^2^ and 0.123 rel. units, respectively. In the presence of MgATP, the E173A mutation decreases the value of Φ_E_ by 2.8° and had a large effect on the bending stiffness of F-actin at the site of localization of the myosin head and on the flexibility of myosin head attachment to F-actin. Indeed, in the presence of MgATP at low Ca^2+^ (mimicking relaxation) the values of ε and N decreased by 43% and 91%, which demonstrated an increased number of myosin heads strongly bound to F-actin, instead of the anticipated increase in the rigidity of F-actin and flexibility of myosin attachment to actin typical for relaxation. It is noteworthy that the bending stiffness is 5.15 × 10^−26^ N∙m^2^ typical for AM state in the presence of WT-Tpm at low Ca^2+^ and the flexibility of the attachment of the myosin heads to actin is much lower than with WT-Tpm (the value of N is 0.042 rel. units; Figure 6e,h and Figure 7), showing “fixation” of the myosin heads on the thin filament. Such changes in these parameters can be interpreted as formation of so-called rigor-like myosin heads in the muscle fibres [7]. The appearance of the rigor-like cross-bridges can not only have a profound effect on relaxation, but also bring about disorganization of the thin and thick filaments. The appearance of these cross-bridges could contribute to the hypotonia and motor delay observed in a patient with the mutant E173-Tpm [17].

Thus, the E173A mutation in Tpm is able to facilitate the strong binding of the myosin heads to F-actin at low Ca^2+^ at different states of the ATPase cycle. This may be the reason for the high Ca^2+^-sensitivity of in vitro actin-activated S1 ATPase (Figure 1).

At high Ca^2+^ the E173A-Tpm mutation causes a small decrease in the number of strongly-bound myosin heads (the Φ_E_ is higher by 0.4° at AM state and by 0.5° at mimicking the AM^∙ADP state, *p* < 0.05; Figure 7). In the AM*∙ADP∙Pi state, the parameters Φ_E_ and N practically do not change (Figure 7). Therefore, the amplitude of change in the values of Φ_E_ at transition of the myosin heads from the weak to the strong binding with F-actin during the ATPase cycle (between the weak binding in the presence of MgATP and the strong binding in the absence of the nucleotides) was 5.52° for E173A-Tpm (Figure 6g), which was smaller than the amplitude observed for WT-Tpm (6.02°) (Figure 6a). It can be assumed that the E173A-Tpm mutation inhibits the efficiency of the cross-bridge work [6,22,24]. This conclusion is consistent with data showing a decrease in the actin-activated ATPase activity of S1 at high Ca^2+^ (Figure 1).

We suggested that replacing the negatively charged glutamate 173 for neutral hydrophobic alanine may cause the salt bridge [25,26] between tropomyosin residues E173 and K169 to break, and as a result, partially destabilize the tropomyosin molecule. It was suggested that the residue 174 of tropomyosin is cross-linked with troponin-T and this residue can participate in tropomyosin interaction with troponin [27]. Therefore, the E173A mutation can both change the stability of the tropomyosin molecule and alter the binding of tropomyosin to troponin-T. In this study no effect was found of E173A mutation on the tropomyosin position in the absence of troponin (Figure 3), whereas in the presence of troponin, the mutant Tpm shifts toward the open position, mimicking all states of the ATPase cycle at high and low Ca^2+^. We suggested that alteration in the binding of the Tpm to troponin-T can be a reason for the violated ability of troponin to switch the thin filaments on and off. This can lead to a decrease in the amount of the strongly-bound myosin heads at high Ca^2+^, an increase in the Ca^2+^-sensitivity, and may induce appearance of the rigor-like myosin heads which strongly bind to F-actin at mimicking relaxation in muscle fibres (Figure 6h and Figure 7). It should be noted that as far as we know in literature there are no data on the ability of the myosin head to form the strong binding with actin in the presence of MgATP. However, in this work and earlier, data were obtained that could be explained by an increased stiffness of S1 binding to actin. Having screened many mutant forms of various tropomyosins, we were able to find only a few mutant forms that showed such an effect (ΔE139 [7] and R91G in Tpm2.2 [28], R168G in Tpm1.1 [20], and A155T in Tpm3.12 [29]). Moreover, such tightly bound S1 molecules appeared only in the presence of the mutant Tpm and disappeared after addition of W7.2.7. Ca^2+^-sensitivity inhibitor of troponin, W7, may weaken the damage induced by the E173A mutation

It has been known that W7 (n-(6-aminohexyl) 5-chloro-1-napthalenesulfonamide, Sigma-Aldrich) binds specifically with high affinity to troponin-C, but does not interact with actin, myosin, or tropomyosin [30], therefore it can be used as a specific inhibitor of calcium activation in skinned fibres from cardiac and skeletal muscles [31,32]. In addition, it was shown earlier that the desensitizer W7 can correct hyper-calcium-sensitivity of sarcomeres induced by a point mutation [33]. Here we tried to use W7 to reduce the disruption of the actin-myosin interaction during the ATPase cycle in the ghost muscle fibres caused by the E173A mutation in Tpm.

**Figure 8:**
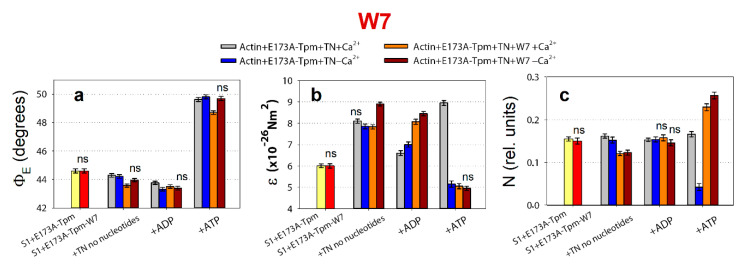
The effect of troponin and Ca^2+^ on the values of Φ_E_ (**a**), ɛ (**b**) and N (**c**) of the polarized fluorescence of 1,5-IAEDANS bound to S1 (AEDANS-S1), in the presence of the E173A-Tpm or E173A-Tpm with 50 μM of W7 revealed in glycerol-skinned fibres under conditions simulating the sequential steps of the actomyosin ATPase cycle. The data represent the mean values for 5–7 fibres for each experimental condition. The Φ_E_, ɛ and N values in the presence of troponin are significantly altered by W7 (*p* < 0.05). Statistically insignificant differences in the values of Φ_E_, ɛ and N between the WT and mutant Tpms are indicated by the symbol “ns”. Error bars indicate ±SEM.

The binding of 50 μM of W7 to Actin-E173A-Tpm-TN-AEDANS-S1 complex induces a change in the values of Φ_E_, ε and N at high and low Ca^2+^ (Figure 8).

At high Ca^2+^, the number of myosin heads strongly bound to F-actin increases (the Φ_E_ values decreases), the bending stiffness (ε) and flexibility of myosin head attachment to F-actin (N) practically do not change, except for a decrease in the presence of MgADP (Figure 8). This means that W7 restores the ability of the myosin heads to bind strongly to F-actin during the ATPase cycle. However, this does not increase the efficiency of the cross-bridge work. Indeed, the amplitude of the changes in myosin conformation during the transition from the weak to the strong binding of myosin heads to F-actin is lower than for WT-Tpm. The amplitude of change in the values of Φ_E_ (from MgATP to no nucleotides) for E173A-Tpm is 5.12° which is smaller than the amplitude observed for WT-Tpm (6.02°) (Figure 7a and Figure 8a).

At low Ca^2+^ W7 does not affect the number of myosin heads strongly bound to F-actin (the values of Φ_E_ practically do not change; Figure 8a), but increases the bending stiffness of F-actin when mimicking the AM and AM^∙ADP states (the values of ε increases by 1.1 × 10^−26^ N∙m^2^ and 1.45 × 10^−26^ N∙m^2^ in the absence of the nucleotide and in the presence of MgADP, respectively; Figure 8b). W7 extremely increases the flexibility of myosin head attachment to actin when modelling muscle fibre relaxation (the value of N increases by 84%; Figure 8c). The latter is very important, since the appearance of the cross-bridges strongly bound with F-actin at relaxation (so called rigor-like myosin heads) can cause contracture and contribute to the development of destructive changes in muscle tissue [34]. Thus, W7 can at least partially restore the balance between the strongly- and weakly-bound myosin heads during the ATPase cycle that is necessary for normal contractility and relaxation in muscle fibres.

Summing up, a major advantage of our in situ structural approach over previous studies in regulation of actin-myosin interaction in protein solution using isolated filaments is that tropomyosin orientation has been determined at physiological conditions and in an intact muscle sarcomere, preserving the native relationship between the myosin and actin filaments. The application of reconstituted muscle fibres has enabled us to reveal unknown details of regulation of actin-myosin interaction by tropomyosin-troponin complex during the ATPase cycle in the muscle fibres, containing the wild-type and mutant E173A tropomyosins. Our data have shown that Ca^2+^ regulation of actin-myosin interaction is mediated by conformational changes in tropomyosin-troponin complex and actin that result in spatial rearrangement and alterations in persistence length of tropomyosin and F-actin (Figure 3b, Figure 5b and Figure 6b,f) that presumably cause azimuthal shifting of the tropomyosin strands [6,7,8]. The conformational changes in troponin-tropomyosin complex and F-actin initiated by Ca^2+^ are interdependent [6], therefore a point mutation in any of these proteins should disrupt this interdependency and induce deregulations of actin-myosin interaction. Our work demonstrates that the substitution E173A induces such uncoupling. Indeed, troponin loses the ability to move tropomyosin strands towards the outer domains of actin and switch actin monomers off at low Ca^2+^ (Figure 3, Figure 4 and Figure 5). That may contribute to the Ca^2+^-dependent changes in the rate of the ATPase and high Ca^2+^ sensitivity that we observed in vitro (Figure 1). In addition, the E173A mutation also may alter the ability of tropomyosin to control the formation of the strong binding of myosin heads to F-actin throughout the ATPase cycle.

We suggest that replacing negatively charged glutamate 173 with neutral hydrophobic alanine may cause the salt bridge between tropomyosin residues 173 and 169 [25,26] to break, and as a result, partially destabilize the tropomyosin molecule in the region of the site for tropomyosin binding to troponin-T. The alteration in the tropomyosin to troponin-T interaction can result in disruption of the ability of troponin to switch the thin filaments on and off. This can lead to inhibition of the ATPase activity at high Ca^2+^ (a decrease in force production) and increase in the Ca^2+^-sensitivity and the appearance of the rigor-like myosin heads which strongly bind to F-actin at relaxation (Figure 7c). Similar myosin heads were observed in our earlier studies of other mutant tropomyosins, which are associated with distal arthrogryposis and cap-myopathy. The so-called rigor-like cross-bridges can be one of the reasons for contracture and disorganization of muscle fibres [7,8]. Therefore, it seems important to reduce the effect of the E173A mutant, for which we used the Ca^2+^-desensitizer W7. It has been shown that W7 restores the ability of troponin to activate the strong binding of the myosin heads to F-actin at high Ca^2+^ and reduces the number of rigor-like myosin heads at relaxation. However, W7 does not restore the ability of troponin to switch the thin filaments off at low Ca^2+^ (Figure 5). Therefore, W7 can be used more likely to reduce the damaging effect of the E173A mutation on muscle contractility.

## 3. Materials and Methods

### 3.1. Use of Experimental Animals

All experiments were performed on skinned muscle fibres and proteins from skeletal muscles of rabbit (*Oryctolagus cuniculus*). The animals were killed in accordance with the official regulations of the community council on the use of laboratory animals by the methods described earlier [6,8]. The study was approved by the Animal Ethics Committee of the Institute of Cytology of the Russian Academy of Science (Assurance Identification number F18-00380, period of validity 12 October 2017–31 October 2022).

### 3.2. Preparation of Proteins and Their Labelling by Fluorescent Probes

Fast skeletal muscle myosin and troponin were isolated and purified by using standard protocols [35,36]. Treatment of myosin with α-chymotrypsin for 20 min at 25 °C yielded S1 free from the regulatory light chains [37]. S1 was modified at Cys707 with 1,5-IAEDANS as described earlier [38]. The recombinant γγ-WT-Tpm (control protein containing no mutations) and the E173A mutant Tpm were obtained using overexpression in *E. coli* BL21(DE3)pLysS and subsequent purification by ion-exchange chromatography, as described earlier [39,40]. The obtained Tpms were stored at –45 °C for several months. The Tpms had an AlaSer N-terminal extension to compensate for the reduced affinity of recombinant non-acetylated skeletal Tpm to F-actin [41]. Tpms were modified at Cys190 with 5-IAF as described previously [6]. The quality of the protein preparations was determined by SDS-PAGE (Figure 2).

### 3.3. Determination of Actin-Activated ATPase of Myosin

The rate of the ATPase reaction was determined for fully regulated reconstituted thin filaments in a solution containing 1 μm S1, 7 μm F-actin, 3 μm troponin, 3 μm WT-Tpm or E173A-Tpm in the following buffer: 12 mm Tris-HCl (pH 7.9), 2.5 mm MgCl_2_, 15 mm KCl, 20 mm NaCl, 0.2 mm dithiothreitol and 2 mm ATP at 25 °C. The reaction was carried out at Ca^2+^ concentrations increasing from 1 × 10^−9^ M to 1 × 10^−4^ M. The concentration of free Ca^2+^ in the presence of 2 mm ethylene glycol-bis(2-aminoethylether)-N,N,N′,N′-tetraacetic acid (EGTA) was calculated using the Maxchelator program (http://maxchelator.stanford.edu/CaEGTA-TS.htm). The reaction was stopped after 10 min by adding trichloroacetic acid to a final concentration of 5%. The amount of inorganic phosphate formed was determined by the method of Fiske and Subbarrow [42]. Three experiments were conducted for each experimental condition. Statistical processing of data, calculation of the pCa_50_ value and plotting was carried out using GraphPad Prism 5.0 software.

### 3.4. Preparation and Labelling of Ghost Fibres

Models of striated muscle fibres, where due to extraction of myosin and the regulatory proteins actin comprised up to 70–80% of the total muscle protein, were used in this work. These models (so-called ghost fibres) were obtained from *m. psoas* of rabbit. The bundles of about 100 fibres were placed into a cooled solution containing 100 mm KCl, 1 mm MgCl_2_, 67 mm K, Na phosphate buffer, pH 7.0, and 50% glycerol. Single fibres were gently isolated from the glycerinated muscle bundle and incubated during 70–90 min in the solution containing 800 mm KCl, 1 mM MgCl_2_, 10 mm ATP, 6.7 mm K, Na phosphate buffer, pH 7.0 [6]. Thin filaments were reconstructed with Tpm (WT-Tpm or E173A-Tpm) and troponin and decorated with S1 by incubating the fibre in a solution containing the corresponding proteins. The proteins that did not bind with F-actin were removed by the washing of the fibre in the same solution without proteins. FITC-phalloidin was dissolved in methanol and conjugated with F-actin of the fibres as described before [6,8].

The final composition of the fibres was examined using 12% SDS-PAGE gels, stained with Coomassie brilliant blue R (Sigma-Aldrich) and scanned in Bio-Rad ChemiDocTM MP Imaging system (Hercules, CA, USA) (Figure 2). Then, 8–10 fibres were applied to each lane. Excess of the proteins was removed by 60 min flushing of the fibres in the washing solution which contained 67 mm K, Na-phosphate buffer, 100 mm KCl and 1 mm MgCl_2_. The ratio of WT-Tpm to the mutant Tpm that bound to actin was determined in 15% gel by Image Lab 6.0. In addition, the ratio of the E173A and WT Tpms was determined using fluorescence microscopy while focusing on the internal areas of the muscle fibre. The intensity measurements did not detect any noticeable differences between the E173A and WT Tpms in their ratio to actin in the thin filaments (data sets are not presented).

### 3.5. Polarized Fluorescence Measurements

Steady-state polarized fluorescence was measured in ghost fibres using a flow-through chamber and a polarized fluorimeter as described before [16]. Fluorescence from the 1,5-IAEDANS-labeled S1 (AEDANS-S1) was excited at 407 ± 5 nm, and from 5-IAF-labelled Tpm (AF-TM) and FITC-labelled actin (FITC-actin) at 489 ± 5 nm; the intensity of the fluorescence (I) was recorded in the range of 500–600 nm. The probes in ghost fibres were excited by a 250 W mercury lamp DRSH-250 [43]. The exciting light was passed through a quartz lens and a double monochromator and split into two polarized beams by a polarizing prism. The ordinary polarized beam was reflected at the dichroic mirror and was condensed by a quartz objective (UV 58/0.80) on a fibre in the cell on the microscope stage. The emitted light from the fibre was collected by the objective and led to a concave mirror with a small hole. After passing through the lens and a barrier filter, the beam was separated by a Wollaston prism into polarized beams perpendicular and parallel to the fibre axis. The intensities of the four components of polarized fluorescence _‖_I_‖_, _‖_I_⊥_, _⊥_I_⊥_ and _⊥_I_‖_ were detected by two photomultiplier tubes [43]. Fluorescence polarization ratios were defined as: P_‖_ = (_‖_I_‖_ − _‖_I_⊥_)/(_‖_I_‖_ + _‖_I_⊥_) and P_⊥_ = (_⊥_I_⊥_ − _⊥_I_‖_)/(_⊥_I_⊥_ + _⊥_I_‖_). The subscripts ‖ and ⊥ designate the direction of polarization parallel and perpendicular to the fibre axis, the former denoting the direction of polarization of the incident light and the latter that of the emitted light.

The experimental data were assessed by a helix-plus isotropic model [43,44,45]. The model is based on the assumption that there are two populations of fluorophores in muscle fibre: the ordered fluorophores in the amount of (1-N), with their absorption and emission oscillators oriented at the angles Φ_A_ and Φ_E_, respectively, relative to the thin filament axis, and the disordered fluorophores in the amount of N (oriented at the magic angle of 54.7°). The number of disordered probes N relates to the mobility of the labelled protein. Motions of the probes relative to the protein are included in the model as the angle γ (the angle between the absorption and emission dipoles). The value of γ is constant for the probes and is assumed to be 17° for 5-IAF bound to tropomyosin, 14° for FITC bound to F-actin and 20° for 1,5-IAEDANS bound to S1 [43]. In this model the thin filament is assumed to be flexible (i.e., the angle θ between the fibre axis and thin filament is not zero). According to the theory of a semiflexible filament, for a filament length L with one end fixed and the other end free, sin^2^θ = 0.87(kT/ε) L. Thus, the bending stiffness (ε) of actin filaments can be estimated from sin^2^θ [45].

Measurements were carried out in the washing buffer in the absence of nucleotides (simulating the AM state of the actomyosin complex) or in the presence of 3 mM ADP or 5 mM ATP, mimicking, respectively, the AM^·ADP and AM**·ADP·Pi states of actomyosin in the ATPase cycle [6,46]. In the experiments with troponin, the solutions additionally contained either 0.1 mM CaCl_2_ or 2 mM EGTA.

Changes in the polarized fluorescence parameters (Φ_E_, ε and N) were considered as reporting on conformational changes in the protein modified with the probe [6,7,8]. The data were obtained from 5−10 fibres (25–50 measurements) for each experimental condition. Statistical significance of the changes was evaluated using Student’s *t*-test, *p* < 0.05.

## Supplementary Materials

Supplementary Materials (The full-length SDS-PAGE gels) can be found at https://www.mdpi.com/1422-0067/21/12/4421/s1.

## Abbreviations

Tpm	tropomyosin
WT-Tpm	wild-type tropomyosin
E173A-Tpm	tropomyosin with the Glu173Ala substitution
TN	troponin
S1	myosin subfragment-1
1,5-IAEDANS	*N*-(iodoacetaminoethyl)-1-naphthyl-amine-5-sulfonic acid
5-IAF	5-iodoacetamidofluorescein
FITC	fluorescein isothiocyanate
W7	(*N*-(6-minohexyl) 5-chloro-1-napthalenesulfonamide)
ADP	adenosine 5′-diphosphate
ATP	adenosine 5′-triphosphate
EGTA	ethylene glycol-bis(2-aminoethylether)-N,N,N′,N′-tetraacetic acid
