# Supplementary material for: Molecular Mechanisms of Muscle Weakness Associated with E173A Mutation in Tpm3.12. Troponin Ca2+ Sensitivity Inhibitor W7 Can Reduce the Damaging Effect of This Mutation
Source: Int J Mol Sci. 2020 Jun 22;21(12):4421. doi: 10.3390/ijms21124421 (PMC7352912; doi:10.3390/ijms21124421)

Molecular mechanisms of muscle weakness associated with E173A mutation in Tpm3.12. Troponin Ca2+ sensitivity inhibitor W7 can reduce the damaging effect of this mutation

Yurii S. Borovikov [1,*], Armen O. Simonyan [2], Stanislava V. Avrova [1], Vladimir V. Sirenko [1], Charles S. Redwood [3] and Olga E. Karpicheva [1]

The full-length SDS-PAGE gels

Supplementary Figure A. (a) The full-length SDS-PAGE gel showing the composition of muscle fibers (lane 1), of ghost fibers reconstituted with the recombinant wild-type (lane 3) and A155T and E173A mutant (lanes 5 and 7) tropomyosins incorporated in thin filaments, and the recombinant tropomyosins preparations per se (lane 2 for the wild-type Tpm, lane 4 for the A155T mutant, lanes 6, 8 and 9 for different loads of the E173A mutant). The bands used in the Figure 2 (lanes 4-8) are framed.

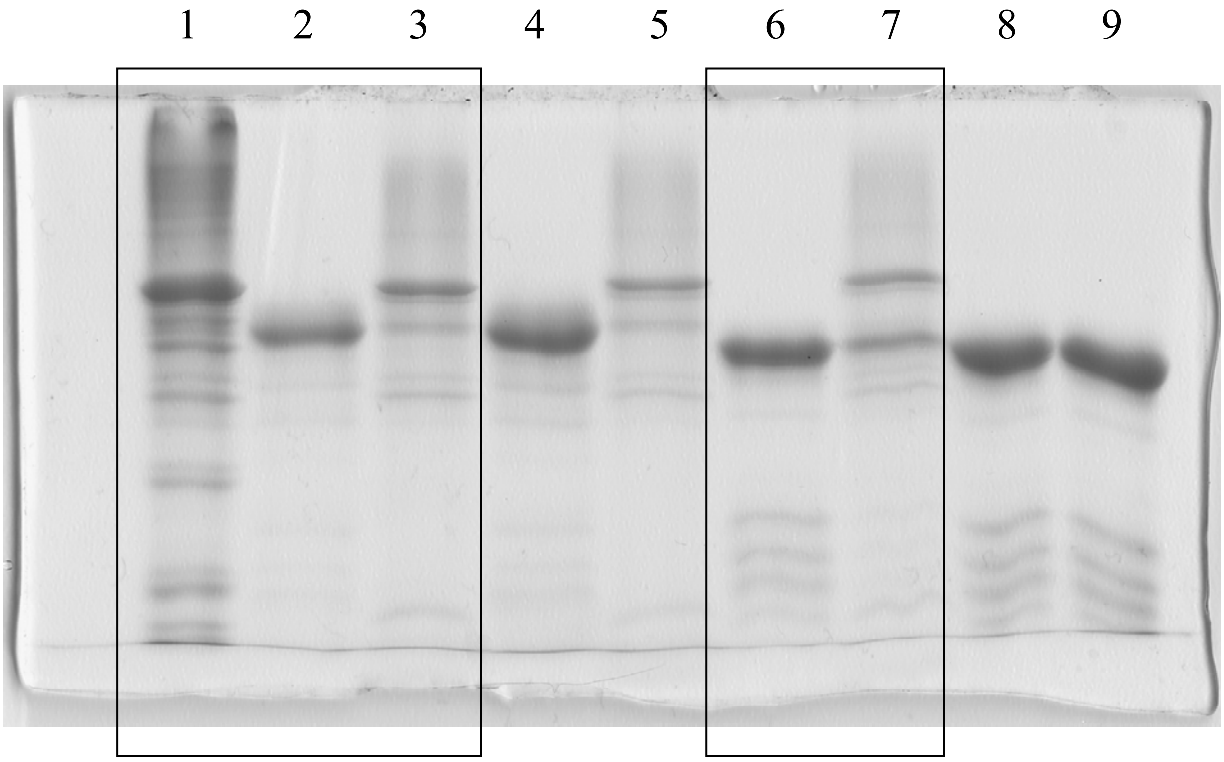

(b) The full-length SDS-PAGE gel showing the results of electrophoretic separation of muscle fiber isolated from *m. psoas* of rabbit (lane 2), fibers after extraction of myosin and regulatory proteins of thin filaments (ghost fibers, lane 3), ghost fibers decorated by myosin subfragment-1 (lane 4) and the recombinant αβ-tropomyosins (lanes 1 and 6), as well as molecular weight markers (Sigma-Aldrich, USA, lane 5). The bands used in the Figure 2 (lanes 1-3) are framed.

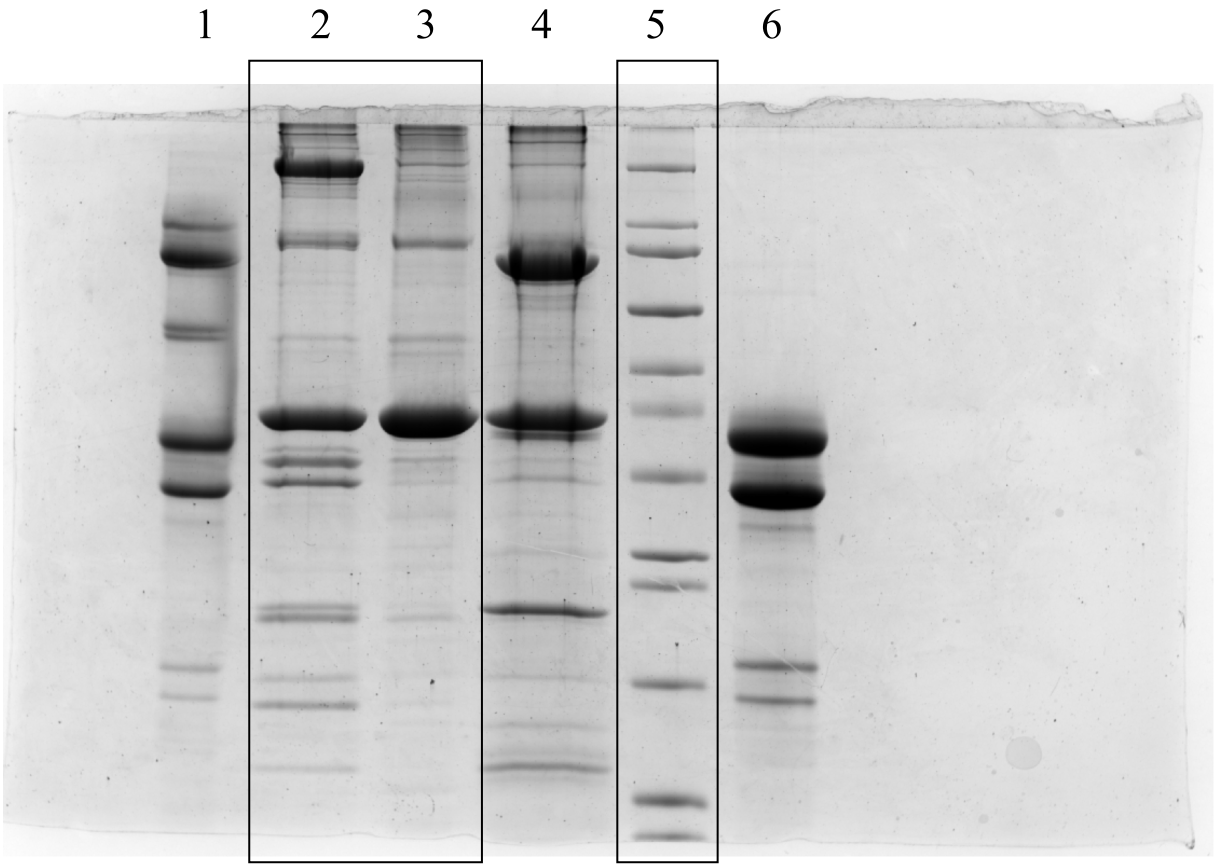

Supplement: Supplementary file 1 [file ijms-21-04421-s001.pdf]
